# Towards an unbiased view of science

**DOI:** 10.1038/ncomms8774

**Published:** 2015-06-22

## Abstract

Authors at *Nature Communications* now have the option to choose double-blind peer review

*Nature Communications* is an open journal and our articles can be read online by all. Our aim is to publish high-quality research across all of the natural sciences, reporting discoveries that are important to specialists within their respective research fields. We welcome scientific submissions from anyone, and we aim to select the papers that we publish based on the significance of the science presented without any bias towards an author's ethnicity, nationality, gender, number of prior publications or any other factor.

For those manuscripts passing our editorial screening process, peer reviewers are involved in assessing papers against editorial and scientific criteria. Although we believe our reviewers judge all manuscripts individually based on their merits, we recognize that as humans there is a potential for subconscious bias despite striving to reach an objective decision. Our editorial processes, including involving experts, can help to reduce such problems, yet no system can ever be perfect.

A widely discussed initiative to limit further bias in the manuscript selection process is double-blind peer review. Our existing peer review system is ‘single-blind', in that authors are unaware of the identity of the reviewers assessing their manuscript. In double-blind peer review, the reviewers are also unable to see the identity of authors, thus removing potential or perceived bias from reviewers against individual authors.

Following on from our *Nature*-branded sister journals, *Nature Communications* is very pleased to offer our authors a double-blind peer review option. We believe that double-blind peer review represents an exciting option for our authors, and welcome submissions from anyone interested in selecting this option.

During double-blind peer review the identity of authors is known to the editors, and some might call for a ‘triple-blind' system to eliminate the potential for editorial bias. This would not only be difficult to implement, it would also come at the cost of preventing direct discussions between editors and the authors they serve. However, most of our submissions are assessed by more than one editor, and decisions after double-blind peer review are, of course, made on the basis of the reports of reviewers who are not aware of the authors' identities. A checklist that might be useful to ensure that author identity is not readily apparent to reviewers can be found at http://www.nature.com/authors/double-blind-checklist.pdf.

Double-blind peer review is not a complete solution to potential bias. For example, experts in a field serving as peer reviewers might be able to guess the identity of authors based on the science contained in the paper, or from what they know about the work from conference talks or posters. We already ask our reviewers to disclose any conflicts of interest when we approach them to review a paper, or if this becomes apparent during the review process. This requirement will remain for manuscripts submitted for double-blind peer review.

Double-blind peer review is only one measure to enhance the objectivity of the publication process. And it will not be the last. We will continue to assess further ways to improve our publication process and to provide the best possible service to our authors as well as to our readers. As with the other *Nature*-branded journals, we very much welcome your input on this initiative.

